# Supplementary material for: The discovery of novel noncoding RNAs in 50 bacterial genomes
Source: Nucleic Acids Res. 2024 Apr 22;52(9):5152–65. doi: 10.1093/nar/gkae248 (PMC11109978; doi:10.1093/nar/gkae248)
Supplement: gkae248_Supplemental_Files [file gkae248_supplemental_files.zip › Narunsky 50 Genomes RNA Motifs Files.zip!/Publication/69-E_rhusiopathiae/Figures/LRC-69-9-NC_015601_1_IGR_33_121491_121884.sto.pdf]

LRC-69-9-NC_015601_1_IGR_33_121491_121884.sto.r2r

5′-A●AAAACCACC●AAUC●GGUGGUUUUUUR●URUUUGUGAUUCCAUUCGA●YURYAUY●GAAGGGAAURUUYUAAUYAAGRACAGGCACAAUUGYCUYRAUUARR●YYYYUGUUGAAAAAGGA●RUUGUGUUUAAUCGAUAACUUAGAURAGAUUAAACR●U●UGAGUAUUAGAAUYAAUYAUCCUACCAAAGAUR●Y●U●AU●CUGAAUUAAAUCAAAAUCUAAUUCACRUA●●UAUUAUAUAGUA●●●U●U●U●RUGUGA●○○A●YAAA●GUUUGA●●●CRCAY○○○○○○○AYU●Y●AYUUG●A●CGUGUU●U●CAY●●A●●●RR●AARUAUGUGAAA●●●●U●UUAAAC●UCCACAAUU●R●AU○○○○○○○○○○○○○○○○○○○○○○○○
